# Supplementary material for: Roadkills as a Method to Monitor Raccoon Dog Populations
Source: Animals (Basel). 2021 Nov 4;11(11):3147. doi: 10.3390/ani11113147 (PMC8614573; doi:10.3390/ani11113147)
Supplement: Supplementary file 1 [file animals-11-03147-s001.zip › animals-1398080-supplementary.pdf]

Supplements

Table S1. Normality of the raccoon dog roadkill index (non-normal cases shaded) and the proportion of null counts by year and month, 2007–2020, in Lithuania.

Year	D	p	PR	Month	D	p	PR
2007	0.29	0.20	0.62	January	0.38	0.29	0.93
2008	0.16	0.13	0.68	February	0.27	0.40	0.91
2009	0.20	<0.001	0.71	March	0.24	0.07	0.84
2010	0.30	0.28	0.60	April	0.25	0.01	0.89
2011	0.34	0.19	0.68	May	0.30	0.002	0.86
2012	0.19	0.66	0.55	June	0.30	0.005	0.89
2013	0.22	<0.001	0.65	July	0.18	0.002	0.75
2014	0.21	<0.001	0.73	August	0.18	<0.001	0.56
2015	0.26	<0.005	0.76	September	0.18	<0.001	0.57
2016	0.19	0.50	0.85	October	0.14	<0.02	0.75
2017	0.30	<0.05	0.82	November	0.24	<0.05	0.69
2018	0.15	0.82	0.92	December	0.33	0.43	0.89
2019	0.20	0.74	0.92				
2020	0.11	0.37	0.85				

D – Kolmogorov-Smirnov’s D, p – probability that distribution is differing from the normal, PR – proportion of the null counts.

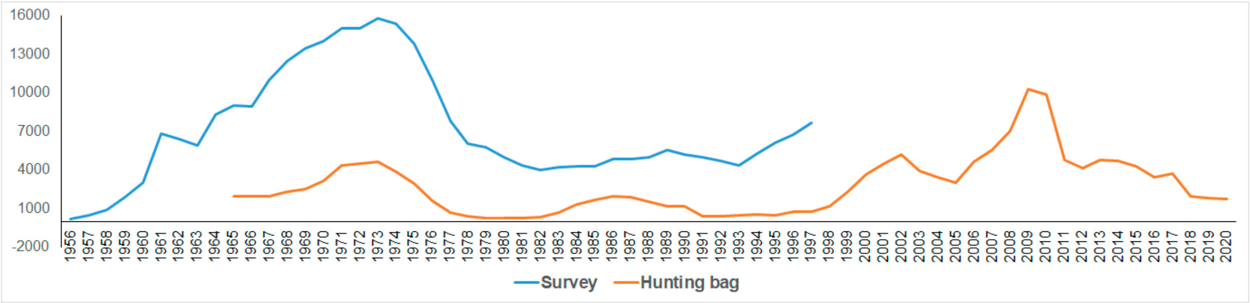

Figure S1. Raccoon dog numbers (1956–1997) and hunting bag sizes (1965–2020) in Lithuania according to the official source, the Ministry of the Environment.

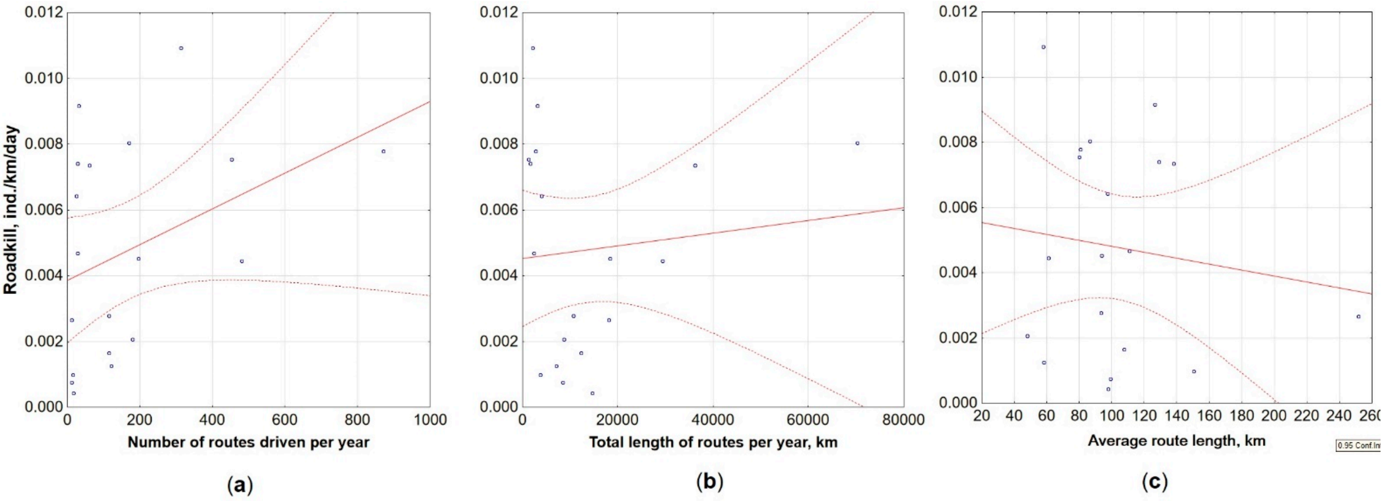

Figure S2. Raccoon dog roadkill index correlations with sampling intensity: (**a**) – number of routes driven per year, (**b**) – total length of routes driven per year, (**c**) – average length per one route driven. Dashed lines show 95% CI.

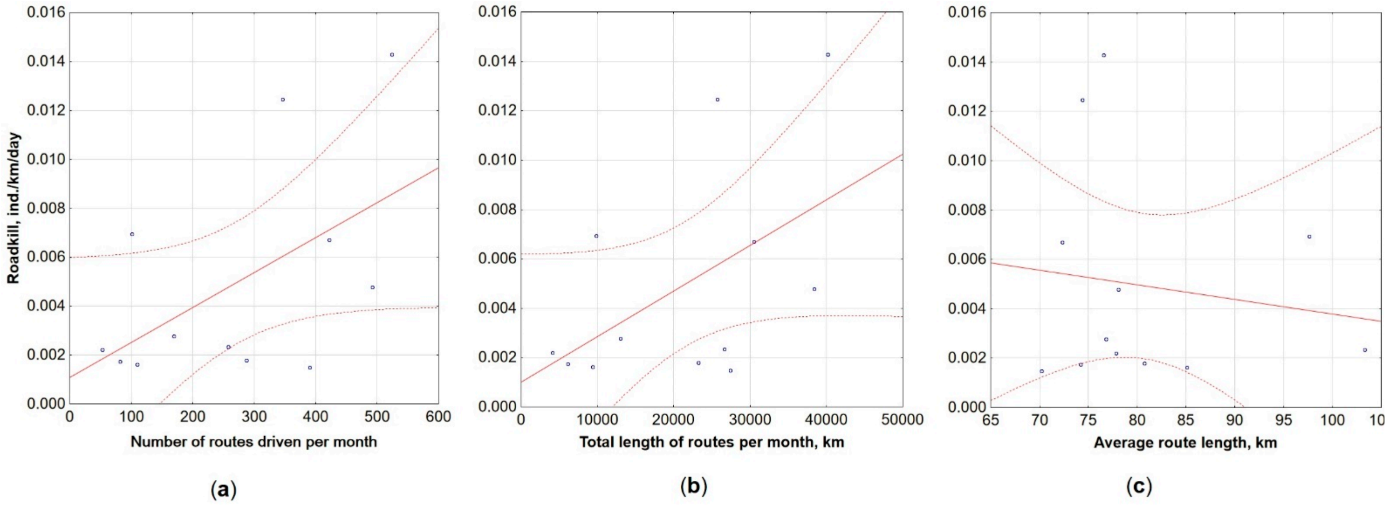

Figure S3. Raccoon dog roadkill index correlations with sampling intensity: (**a**) – number of routes driven per month, (**b**) – total length of routes driven per month, (**c**) – average length per one route driven. Dashed lines show 95% CI.

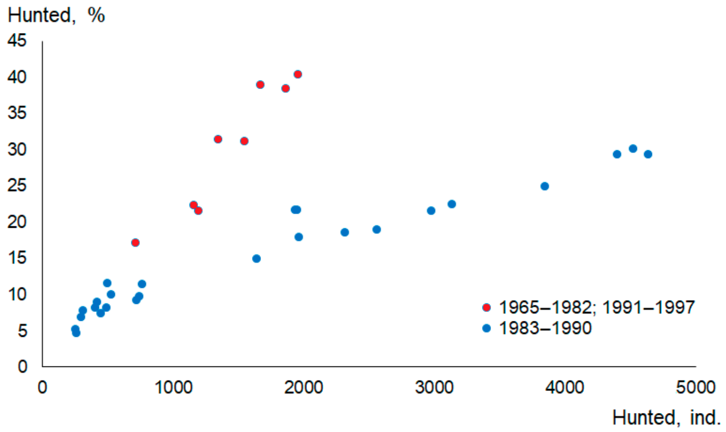

Figure S4. Changes in the number of hunted raccoon dog individuals and the proportion of the hunting bag from the population size in 1965–1997.

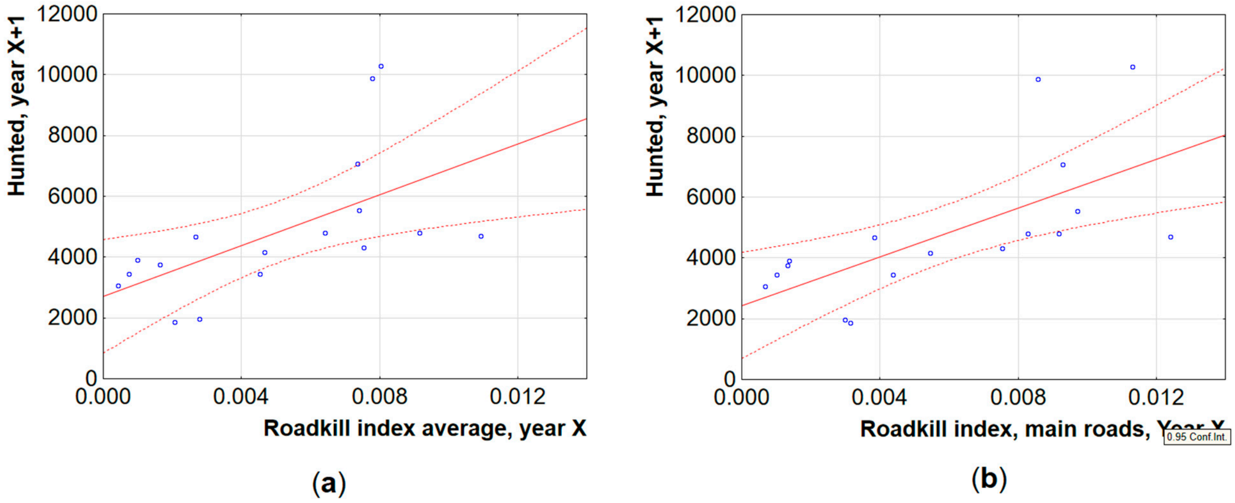

Figure S5. Prognostic value of the average raccoon dog roadkill index (**a**) and roadkill index on the main roads (**b**) towards hunting bag size in the next year, 2002–2020. Dashed lines show 95% CI.

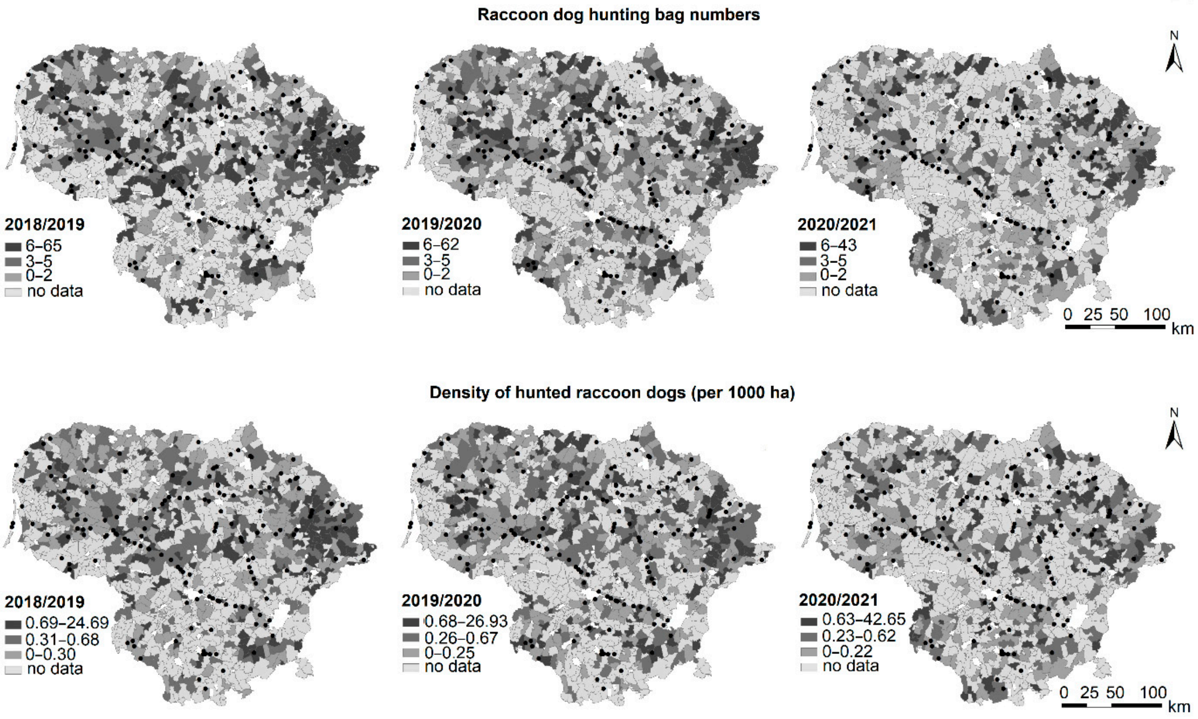

Figure S6. Yearly spatial distribution of raccoon dog hunting bag numbers and the density of hunted animals in 2018 to 2020 at the local scale. White colour denotes territories of towns and cities, excluded from the hunting areas; dots represent raccoon dog roadkills in 2018–2020.

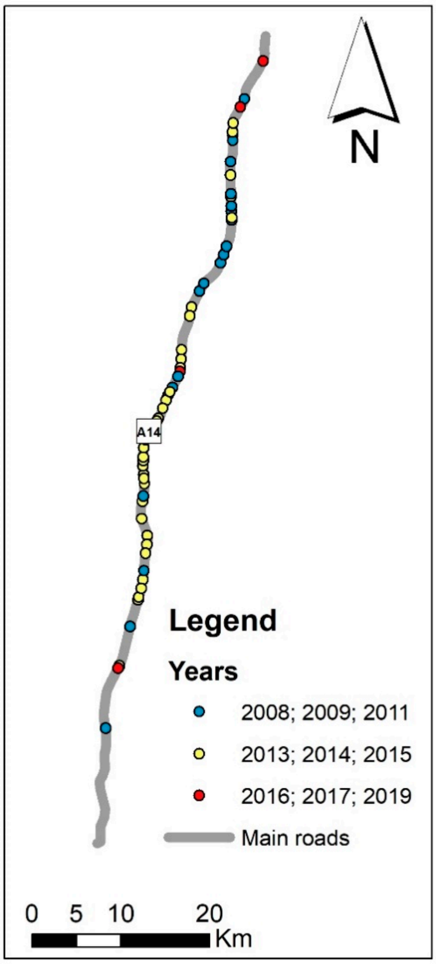

Figure S7. Spatial distribution of the roadkills of raccoon dogs on the A14 main road in 2007–2019. Location of the A14 is shown in Figure 1.
